# Reconstruction of the near-field distribution in an X-ray waveguide array^1^

**DOI:** 10.1107/S1600576717004630

**Published:** 2017-05-16

**Authors:** Qi Zhong, Lars Melchior, Jichang Peng, Qiushi Huang, Zhanshan Wang, Tim Salditt

**Affiliations:** a Institut für Röntgenphysik, Universität Göttingen, Friedrich-Hund-Platz 1, 37077 Göttingen, Germany; b MOE Key Laboratory of Advanced Micro-Structured Materials, Institute of Precision Optical Engineering, Department of Physics, Tongji University, Shanghai 200092, People’s Republic of China

**Keywords:** X-ray optics, X-ray waveguides, nano-focusing waveguide arrays

## Abstract

Iterative phase retrieval has been used to reconstruct the near-field distribution behind tailored X-ray waveguide arrays, by inversion of the measured far-field pattern recorded under fully coherent conditions. It is thereby shown that multi-waveguide interference can be exploited to control the near-field distribution behind the waveguide exit. This can, for example, serve to create a secondary quasi-focal spot outside the waveguide structure. For this proof of concept, an array of seven planar Ni/C waveguides are used, with precisely varied guiding layer thickness and cladding layer thickness, as fabricated by high-precision magnetron sputtering systems. The controlled thickness variations in the range of 0.2 nm results in a desired phase shift of the different waveguide beams. Two kinds of samples, a one-dimensional waveguide array and periodic waveguide multilayers, were fabricated, each consisting of seven C layers as guiding layers and eight Ni layers as cladding layers. These are shown to yield distinctly different near-field patterns.

## Abstract 2

Multi-waveguide interference can be verified experimentally by reconstructing the near-field from the measured far-field diffraction pattern. This enables a direct visualization of the near-field interference pattern and the diversity of fields that can be created by multi-waveguide design, in particular a secondary quasi-focal spot. Numerical propagation using the design parameters is compared with the phase retrieval results.

## 1. Introduction   

X-ray waveguides (WGs) enable manipulation of X-ray fields at the nanoscale, based on the optics of guide modes. Similarly to their optical counterparts, they enable optical functions such as collimation, mode selection and coherence filtering (Osterhoff & Salditt, 2011 ▸) as well as beam splitting for interferometry (Fuhse *et al.*, 2006 ▸), beam tapering (Chen *et al.*, 2015 ▸) and angular redirections (Salditt, Hoffmann *et al.*, 2015 ▸). With typical diameters *d* of the guiding core in the range of a few tens of nanometres, they also form suitable quasi-point sources for X-ray holography (Bartels *et al.*, 2015 ▸). For this application, the length of the waveguide *L* has to be sufficiently long to absorb all radiative modes in the cladding, requiring *L* to be in the range between 0.1 and 10 mm, depending on the photon energy. Generally, one distinguishes between one-dimensionally confining planar waveguides (Spiller & Segmüller, 1974 ▸; Feng *et al.*, 1993 ▸; Lagomarsino *et al.*, 1997 ▸; Zwanenburg *et al.*, 1999 ▸; Jark & Fonzo, 2004 ▸; Egorov & Egorov, 2001 ▸) and two-dimensionally confining channel waveguides (2DWGs), which were introduced by Pfeiffer *et al.* (2002 ▸), and which require advanced electron lithography with interferometric positioning and suitable pattern transfer techniques in order to reach the required aspect ratios. The fabrication of 2DWGs was improved by Fuhse & Salditt (2005 ▸) and more recently extended from overgrown polymer channels to air channels capped by wafer bonding techniques (Neubauer *et al.*, 2014 ▸; Chen *et al.*, 2015 ▸). In this form 2DWGs now serve as fully operational secondary sources for holographic imaging (Bartels *et al.*, 2015 ▸). Notwithstanding this successful development, lithography still lacks the precision to which planar thin films can be fabricated. Therefore, for purposes of highest beam confinement or to exploit novel geometries, wave guiding in only one dimension as in thin planar films is suitable, owing to the better control of layer sequences that this allows. For example, in this way the theoretical limits for beam collimation (Bergemann *et al.*, 2003 ▸), notably 8 nm for the given material, could be reached in a planar thin-film waveguide with an optimized cladding material (Mo/C/Mo embedded in Ge; Krüger *et al.*, 2010 ▸, 2012 ▸).

X-ray waveguide optics can be generalized from a single guiding film to an array of planar waveguides, enabling more optical functions. For example, using several planar waveguides can serve to increase the coupling efficiency, *i.e*. to collect more incoming beam intensity by a larger effective entrance cross section. Further, with an optimized material combination, the overall transmission and mode structure could be modulated. Finally, interference and coupling effects between the different guiding layers can be exploited. The generic aspects of coupling X-ray waveguide modes are analogous to other forms of coupled resonators, *i.e.* mode splitting, lifting of degeneracies and ultimately – when increasing the number of guides – the formation of a quasi-continuous spectrum of propagation constants analogous to a band structure. This was first demonstrated by Pfeiffer *et al.* (2000 ▸), using a planar thin-film structure with several planar waveguides, placed in proximity to achieve strong coupling of modes. In these experiments, the collimated synchrotron beam was coupled into the array of waveguides *via* the so-called resonant beam coupling scheme through the top of the multilayer structure. In other applications (Prudnikov, 2003 ▸, 2005 ▸), the cladding layer instead of the guiding layer was generalized to a multilayer, while keeping only a single guiding layer. In this way, the internal reflection angles of guided beams could be increased. Periodically structured claddings could also be useful to realize other coupling geometries, namely Bragg couplers. Recently, we have introduced a further multilayer concept to X-ray waveguide optics, which we denote as a waveguide array (WGA) (Zhong *et al.*, 2017 ▸). A WGA consists of an array of planar waveguides with individually tailored guiding layer thickness and hence propagation constants. Further, the individual guides are separated at distances large enough to avoid coupling. We have proposed this novel scheme to achieve special multi-beam interference patterns outside the waveguide after coupling out a number of beamlets with tailored phase and position.

In contrast to the waveguides introduced by Pfeiffer *et al.* (2000 ▸), the WGA must be operated in front-coupling geometry. After coupling of the beam into the front side, the radiation is guided in the multiple waveguides, before the beamlets are finally coupled out at the other side of the structure. Spurious reflected or transmitted beams are removed, since the waveguides are embedded in a non-transparent cladding. Importantly, by variation of the guiding layer thickness 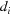 for each waveguide *i* individually, the phase in the exit plane of the waveguide is controlled for each waveguide beamlet individually. In this way, the phase relations between the different guided beams can be tailored to produce special near fields behind the WGA’s exit by multi-waveguide interference (Zhong *et al.*, 2017 ▸). Hence, near-field intensity distributions with special properties can be realized, *e.g.* creating a secondary quasi-focal spot in the free space. For example, in our previous work we used seven planar waveguides with precisely designed layer thickness variations, fabricated by high-precision direct-current magnetron sputtering of carbon (C) and molybdenum (Mo), with systematic thickness variations of the order of 0.2 nm. To this end, the design of the structure must be guided by numerical simulations of field propagation, notably finite-difference (FD) simulations, which predict a beam intensity maximum with a spot size (FWHM) in the sub-50 nm range located in free space behind the WGA at 19.9 keV hard X-ray energy.

In the present paper we show that multi-waveguide interference as introduced by Zhong *et al.* (2017 ▸) can actually be verified experimentally by reconstructing the near-field from the measured far-field diffraction pattern, on the basis of iterative phase retrieval algorithms. Contrary to the approach of Zhong *et al.* (2017 ▸), where the far-field distribution was simulated by using a precise layer combination in a WGA model, the present work enables a much more direct visualization of the near-field interference pattern and a better comparison with the theoretical design. To illustrate the specific field modulating effects which can be achieved by a systematic variation of the waveguide width 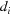 for each waveguide *i*, we have investigated the near field of two different kinds of waveguide structures, namely the aforementioned waveguide array (WGA) and – for comparison – a simpler periodic waveguide multilayer (WGM). The WGA has tailored width 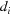 (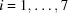) and corresponding cladding layer thickness 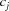 (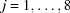) and 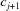 for each waveguide, designed for particular interference effects [quasi-focus, double focus *etc.*, as discussed by Zhong *et al.* (2017 ▸)]. In contrast, the WGM is a periodic arrangement of the same waveguide structure with constant guiding layer *d* and cladding layer *c*. To some extent, the WGM can be regarded as a control sample for the WGA. In both cases, the examples given are structures with a total of 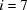 and 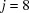 layers, and the guiding layer was composed of amorphous C, while the cladding layer was made of polycrystalline Ni (Zhong *et al.*, 2017 ▸).

With respect to our earlier work (Zhong *et al.*, 2017 ▸), two major experimental steps forward have enabled the successful field reconstruction presented here. Firstly, we have extended the synchrotron experiment from partially coherent bending magnet radiation to highly brilliant undulator radiation (with substantially higher spatial coherence). Secondly, we use a pre-focused beam so that the field is confined in the *xy* plane perpendicular to the optical axis **z**. Note that the phase problem in one-dimensional geometries is generally not amenable to phase retrieval by iterative algorithms (non-uniqueness). Therefore, the changes both in support (focused in *xy* rather than extended in **y**) and in geometry (two-dimensional diffraction pattern rather than line scan) were instrumental. Specifically, the near-field distributions for the WGA and WGM are retrieved from the experimental far-field pattern by using the error-reduction algorithm (Fienup, 1978 ▸, 1982 ▸; Krüger *et al.*, 2010 ▸). The complex-valued field distribution in the exit *xy* plane (amplitude and phase) can then be propagated along the *z* axis and can be compared with the FD calculations of the designed WGA parameters.

The paper is organized as follows. §2 describes the design of the Ni/C WGA and optical field simulations. §[Sec sec3]3 describes the fabrication and characterization of the transmission electron microscopy (TEM) samples. §4 then presents the experimental parameters and results, leading to the near-field reconstruction, before the paper closes with a brief summary and outlook in §[Sec sec5]5.

## 2. Design and simulations   

**Figure 1:**
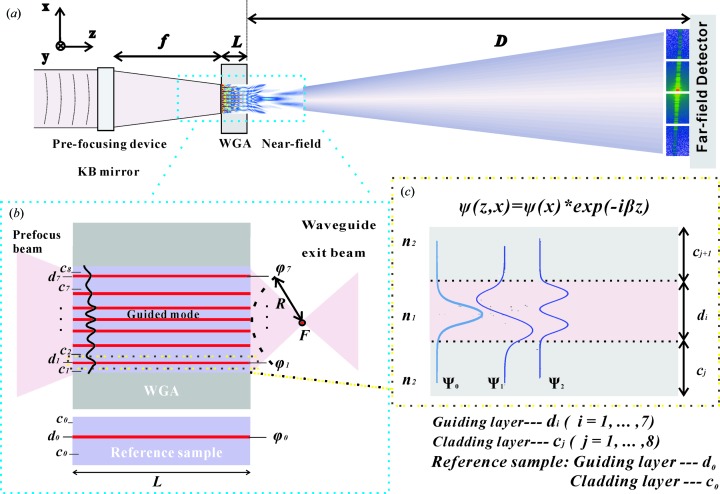
(*a*) Schematic of the experimental setup. The X-ray waveguide array (WGA) is positioned at *f*, which is the distance from the exit of the Kirkpatrick–Baez (KB) device to the sample. The incoming beam with photon energy *E* and primary intensity *I*_0_ is coupled into the Ni/C WGA with working length *L*, which tailors the near field to the desired shape. The far-field intensity distribution is recorded at a distance of *D* behind the WGA exit by a two-dimensional pixel detector. (*b*) A schematic of the structure of the WGA, consisting of seven guiding layers in red (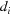, 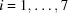) and eight cladding layers in purple (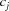, 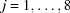). After the pre-focus beam has been coupled, the guided mode is produced in the different guiding layers. With the working length *L*, the exit phase 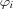 from the corresponding guiding layers *i* can be controlled by the variation of the layer thickness 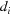. The parameters at the exit of the WGA can be optimized such that the lines of the exit phase 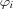 describe a circle with radius *R*, resulting in constructive interference in a quasi-focal spot (*F*) outside the WGA. The phase of the reference sample with length *L* is 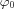, with the corresponding guiding layer 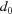 and cladding layers 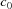. (*c*) Sketch of a slab waveguide with two cladding layers 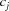 and 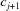. Under the influence of the electric field inside the waveguide [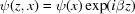], the symmetrical guided modes (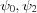) and the asymmetrical mode 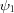 propagate inside the guiding layer depending on the different layer thickness 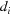.

The WGA is designed to work as a front-coupled waveguide, as illustrated in Fig. 1 ▸(*a*). The synchrotron beam is coupled in, guided in the set of parallel planar layers and then coupled out, to yield the desired near-field pattern in the free space behind the WGA. The exit beam is subsequently broadened again by diffraction and finally diverges to the far-field pattern, which is the main experimental observable. Before addressing the WGA structure designed in this work, we first repeat the basic optical concept of the WGA. The incoming beam of photon energy *E* and primary intensity *I*_0_ is coupled into the Ni/C WGA with working length *L*. The WGA tailors the near field to the desired shape, *e.g*. forming a quasi-focal spot. The two-dimensional far-field intensity distribution is recorded at a distance of *D* behind the WGA exit by a two-dimensional detector. As shown in Fig. 1 ▸(*b*), the WGA, consisting of seven guiding layers (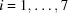) shown in red and eight cladding layers (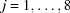) in purple, produces a guided mode in each guiding layer *i*. Let us briefly consider the beam propagation in a slab waveguide *i* with working length *L* and initial guiding layer thickness 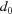. The guiding layer (C) thickness is 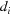, and the thicknesses of the adjacent two cladding layers (Ni) are 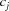 and 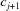 as shown in Fig. 1 ▸(*c*). The refractive indices of the guiding and cladding layers are 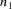 and 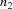, respectively.

The field in the waveguides can be calculated by the reduced Helmholtz equation (Marcuse, 1974 ▸; Osterhoff & Salditt, 2009 ▸), 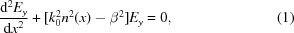
where β is the propagation constant, and the magnitude of the wavevector **k** in the **z** direction is given as 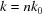 in the corresponding medium. For 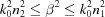, the solution of equation (1) for symmetrical modes can be written as 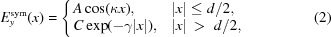
where the parameters of the solution are linked to β according to 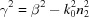 and 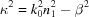. Continuity at the interfaces then leads to a discrete set of solutions, which are determined from the transcendental equations 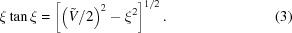
Here, the wave parameter is 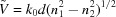, and the propagation constant is 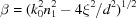. After a Taylor series expansion (Zhong *et al.*, 2017 ▸), the relationship between the propagation constant β and the guiding layer thickness *d* for symmetrical modes becomes 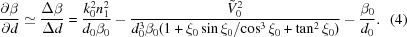
Therefore, the propagation constant β and hence the phase of the exit beam can be controlled by variation of the guiding layer thickness. For the experimental materials and parameters 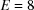
 keV and 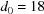
 nm, we obtain 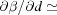 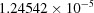. To optimize the thickness values of the seven guiding layers (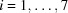), we first determine the required exit phase 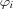 as presented in our earlier work (Zhong *et al.*, 2017 ▸). The phase of a beamlet from the reference sample, as shown in Fig. 1 ▸(*b*), is 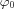 for constant *L* and a given (initial) guiding layer thickness 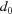. Physically, only the relative phase differences 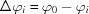 matter for the near-field distribution. The propagation constants then follow from 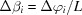. Finally, with the corresponding slight changes in the guiding layer thickness [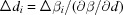], the seven guiding layer thicknesses of the WGA (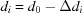) are calculated. After propagating over a distance *L* in the WGA, the value of the exit phase 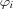 is thus determined by the corresponding guiding layer 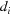. Note, however, that the numerical simulations presented below are not using this approximation.

This phase and the layer positions are the main parameters to optimize and design specific near-field distributions. The parameters of the WGA can be optimized such that the lines of the exit phase 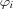 describe a circle with radius *R*, as shown in Fig. 1 ▸(*b*), which results in a quasi-focal point *F* in the near field. When the initial guiding layer thickness (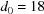
 nm) is set, *R* is mainly influenced by the cladding layer thickness 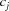. With increasing 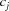, the interference point *F* moves farther away from the exit plane of the WGA. From this analysis, it appears that several interesting refraction and interference phenomena can occur in the WGA structure and free space, and can easily be controlled by changing the cladding layer thickness 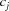.

**Figure 2:**
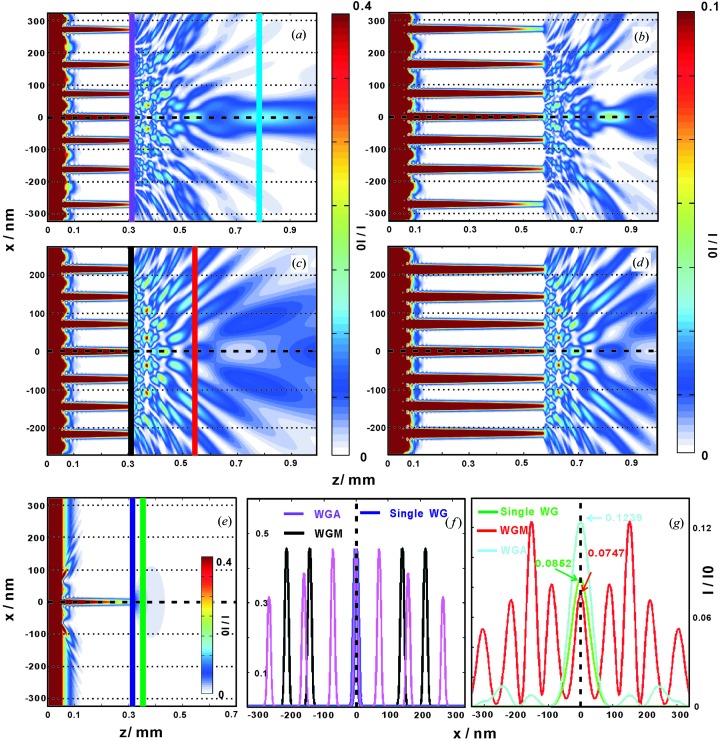
On the basis of the design parameters from Table 1 ▸, field propagation in the WGA and the WGM were simulated in the near field by FD calculations, with the incoming plane wave of unit intensity and 13.8 keV photon energy. The simulations are for waveguide lengths 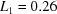
 mm [(*a*) and (*c*) for the WGA and WGM, respectively] and 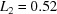
 mm [(*b*) and (*d*), respectively]. (*e*) The field distribution of a single WG (Ni [52 nm] / C [18 nm] / Ni [52 nm]) on a Ge substrate are also calculated for the length 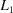. (*f*) The intensity profiles in the exit plane for the WGA [purple line, (*a*)], the WGM [black line, (*c*)] and the single WG [dark-blue line, (*e*)] are compared. (*g*) Comparison of intensity profiles in the downstream planes, for the WGA (light-blue line) at a distance of 0.48 mm from the exit, for the WGM (red line) at a distance of 0.22 mm from the exit, and for the single WG (green line) at a distance of 0.02 mm from the exit. The corresponding intensities 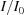 of the WGA, the WGM and the single layer at the central positions are 0.1239, 0.0747 and 0.0852, respectively.

To illustrate the field modulating effects that can be achieved by a systematic variation of the waveguide width 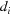, we have simulated the near fields for two different kinds of waveguide structures, WGA and WGM. Both consist of seven C layers and eight Ni layers. Using the different guiding layer thickness 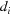 and cladding layer thickness 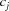 to control the exit phase 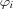 in the WGA, a quasi-focal spot *F* can be created as introduced by Zhong *et al.* (2017 ▸). To emphasize the controlled phase 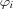 in the WGA structure, a simple periodic structure WGM is used as a reference, with parameters given in Table 1 ▸. From the FD calculations presented by Fuhse & Salditt (2005 ▸), the electromagnetic fields inside the WGA and WGM are simulated for an X-ray energy of 13.8 keV. We perform simulations for the waveguide lengths 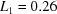
 mm (Fig. 2 ▸ *a* and 2 ▸ *c*) and 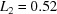
 mm (Fig. 2 ▸ *b* and 2 ▸ *d*) for the WGA and WGM, respectively. In the case of the WGA, the relative intensity 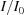 and FWHM of the quasi-focal spot are 0.16870 and 22.0 nm in Fig. 2 ▸(*a*) for 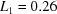
 mm, whereas 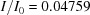 and the FWHM is 25.8 nm in Fig. 2 ▸(*b*) for 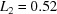
 mm. Compared to the Mo/C WGA considered in our earlier report (Zhong *et al.*, 2017 ▸), where we presented simulations with a quasi-focal spot of FWHM 37.2 nm, located 180.0 µm behind the exit, the Ni/C WGA used in the present work exhibits a higher numerical aperture and a more desirable near-field distribution owing to the variations in cladding layer thickness 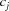, yielding a focal spot at 224.6 µm behind the device, with an FWHM of 22.0 nm. The field distribution in free space behind the WGM is quite similar for the different optical lengths 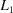 (Fig. 2 ▸ *c*) and 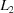 (Fig. 2 ▸ *d*). Hence, the length of the WGM is not as important as the layer structure itself. For comparison, we also present the field distribution of a single WG (Ni [52 nm] / C [18 nm] / Ni [52 nm]) on a Ge substrate (see Fig. 2 ▸ *e*). The corresponding one-dimensional intensity profiles for the WGA, WGM and single WG are plotted in the exit plane and a downstream plane in Figs. 2 ▸(*f*), and 2 ▸(*g*), respectively. For the WGA, a quasi-focal point with the intensity 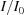 = 0.1239 at a distance of 0.48 mm from the exit is observed.

## 3. Fabrication and characterization   

To evaluate the performance of a WGA, two kinds of structures have been fabricated. First, a prototypical WGA with the characteristic variations in the guiding layer thickness 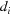 and the corresponding cladding layer thickness 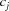. Second, a simple control structure (WGM) with fully periodic waveguide layers, *i.e.* with constant *d* and *c*. In both cases the guiding layer is composed of amorphous C and the cladding layer of polycrystalline Ni, following the parameters shown in Table 1 ▸. The 15 layers for each kind of sample (WGA and WGM) were deposited by direct-current magnetron sputtering (Zhong *et al.*, 2012 ▸, 2013 ▸) at the Institute of Precision Optical Engineering at Tongji University, China. The seven C layers and eight Ni layers were deposited on Si substrates alternately, under a base pressure of 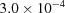
 Pa. The sputter gas was Ar with a purity of 99.999%, and the gas pressure was kept constant at 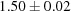
 mTorr (0.1995 Pa). The bonding process was carried out after the fabrication, following Krüger *et al.* (2012 ▸). The structures were bonded to an Si wafer, by an In52Sn48 alloy layer (GPS Technologies GmbH, indalloy number 1E), and using a vacuum oven at 523 K for one hour, keeping the base pressure at 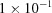
 Pa. Afterwards, the WGA was sliced into 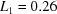
 mm thick samples and the WGM was sliced into 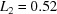
 mm thick samples, ready for the synchrotron experiments. These were carried out at the GINIX (Goettingen Instrument for Nano-Imaging with X-rays) experimental setup, installed at the P10 beamline at the PETRA III synchrotron facility in Hamburg (DESY). The far-field diffraction patterns of the waveguided beams leaving the structures were recorded by an Eiger 4M pixel detector (Dectris). The X-ray energy was set by an Si(111) channel cut monochromator to 13.8 keV. The setup is described in detail by Salditt, Osterhoff *et al.* (2015 ▸). In the experiment, the focusing of the synchrotron radiation by the KB mirrors has to match such that the focal spot size is larger (but not very much larger) than the WGA, which is 658.00 nm (designed structure as shown in Table 2 ▸). With fully opened entrance slits in front of the KB mirrors, the beam size at GINIX was around 295 × 181 nm in the **x** and **y** directions. Therefore, experiments were carried out with smaller slits, notably with a 50 µm slit size, to achieve a spot size broadened by diffraction [see also the ptychographic probe reconstructions presented by Wilke *et al.* (2014 ▸)]. Moreover, this setting ensures full spatial coherence.

**Figure 3:**
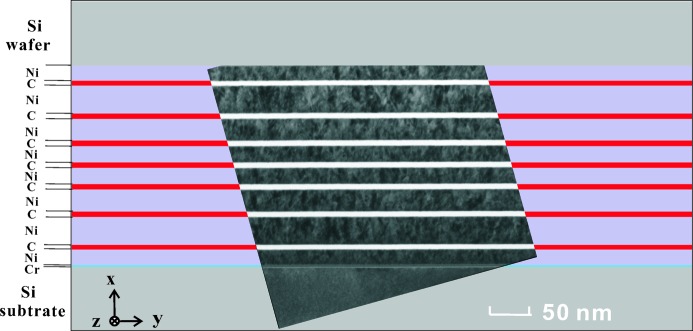
The TEM images of the cross section of the multilayer with seven C guiding layers and eight Ni cladding layers in the WGA structure, bonded to an Si cap wafer. Scale bar 50 nm.

Compared to the periodic structure of the WGM, the layer parameters of the WGA are more critical and therefore have to be precisely characterized before the synchrotron experiments, in order to verify whether the design parameters have been reached (Zhong *et al.*, 2017 ▸). To this end, TEM (using a Philips CM 200 FEG-UT instrument) was used to determine the layer thicknesses for slices cut out by a focused ion beam (FEI Nova Nanolab 600). Several transmission electron micrographs were acquired with partial overlap to cover the WGA cross section (see Fig. 3 ▸). The scale bar represents 50 nm and the pixel size is 0.45 nm. The averaged layer thickness values in several micrographs over 52 line cuts of different parts of the TEM specimen were calculated, with error bars of ±0.45 nm, as shown in Table 2 ▸.

## 4. Results   

**Figure 4:**
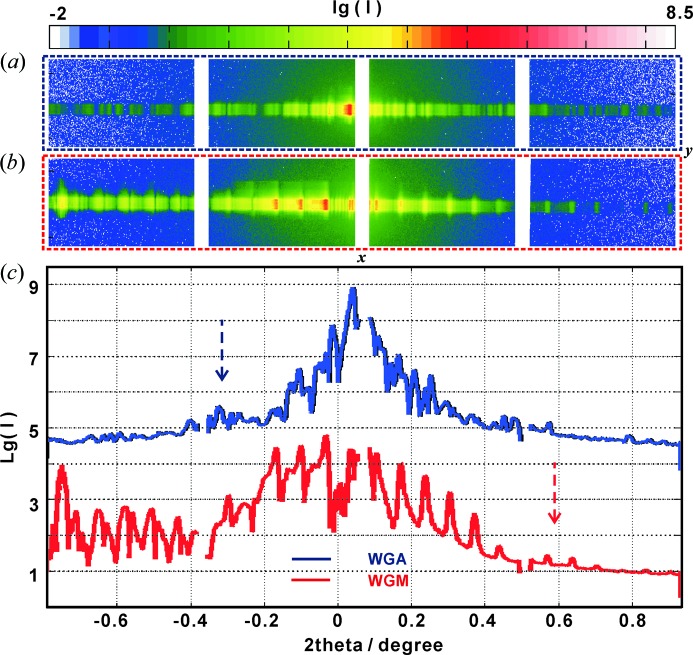
The measured two-dimensional far-field pattern of the WGA (*a*) and WGM (*b*) at 13.8 keV, recorded with the Eiger 4M pixel detector at a distance of 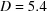
 m behind the structures. (*c*) The integrated one-dimensional far-field curves, corresponding to (*a*) and (*b*).

Fig. 4 ▸ presents the measured far-field patterns of the WGA and WGM, on a logarithmic scale, as recorded with the Eiger 4M pixel detector (Dectris), with pixel size 75 µm, placed at 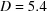
 m behind the focal plane of the KB mirrors. With an X-ray energy of 13.8 keV, the Si wafers of both the WGA 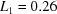
 mm and the WGM 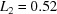
 mm samples are semi-transparent, so that besides the waveguide exit beam there is also a contribution of the primary beam. To minimize this contribution, the detector was aligned such that the primary beam fell onto the inter-module gaps of the detector (with additional attenuation of the beam), as shown in Figs. 4 ▸(*a*) and 4 ▸(*b*), well separated from the extended multilayer signal (vertical stripes). The total accumulation time for the two-dimensional far-field pattern was 10 s, distributed over ten frames. We see that the signal of the WGA is distinctly different from that of the WGM, which exhibits the expected periodic diffraction orders, extending over the entire detector. To better compare the differences of the two structures, the two-dimensional far-field patterns of WGA and WGM were integrated in the **y** direction to yield the corresponding one-dimensional profiles [see, respectively, the blue and red curves in Fig. 4 ▸(*c*)]. In both one- and two-dimensional representations, the ‘grating’ character of the WGM becomes apparent, representing a regular and periodic far-field pattern.

**Figure 5:**
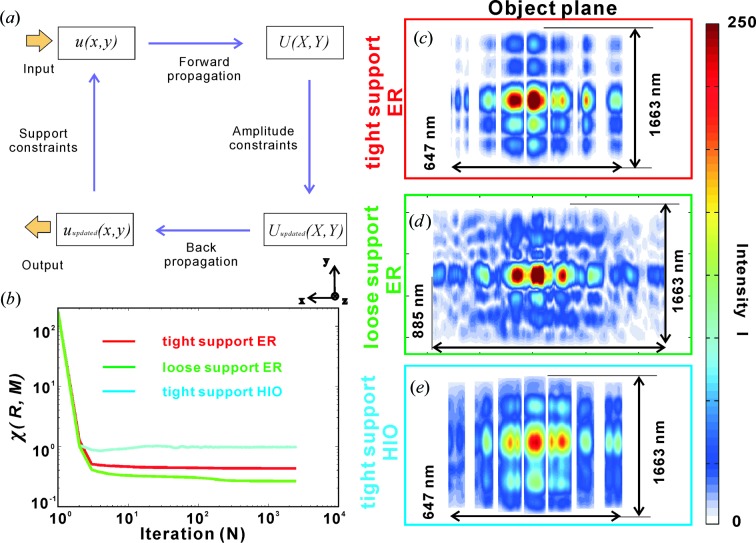
(*a*) Schematic of the iterative reconstruction scheme. (*b*) Error metrics for the tight support using the ER algorithm (red line), the loose support using the ER algorithm (green line) and the tight support using the HIO algorithm (light-blue line). The reconstructed wavefronts are shown after *N* = 2500 iterations, for (*c*) the tight support using the ER algorithm (size 647 × 1663 nm in the **x** and **y** directions), (*d*) the loose support using the ER algorithm (size 885 × 1663 nm in the **x** and **y** directions) and (*e*) the tight support using the HIO algorithm (size 647 × 1663 nm in the **x** and **y** directions).

To further corroborate the correct optical functioning of the WGA, we perform a reconstruction of the complex-valued near-field distribution from the measured far-field pattern (two dimensional), using two different well established phase retrieval algorithms (Elser, 2003 ▸; Marchesini, 2007 ▸), the error reduction (ER) algorithm and the hybrid input–output (HIO) algorithm (Fienup, 1978 ▸, 1982 ▸). Fig. 5 ▸(*a*) illustrates the procedure of the iterative reconstruction scheme. The algorithm is initialized with a guess of the wavefield in the object plane (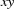). The iteration consists of (i) forward propagation (implemented numerically by a fast Fourier transform) to the far-field detector plane (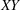 plane), where the wavefield 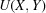 is subjected to an amplitude constraint (measured data), resulting in 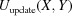, followed by (ii) back-propagation to the object plane, where the field 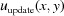 is projected onto the support, resulting in the next input of the cyclic iteration. The experimental parameters, namely the 2167 pixels along the wide direction of the Eiger detector, the pixel size on the detector 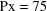
 µm, the detector distance *D* = 5.4 m and the wavelength 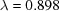
 Å, resulted in a pixel size in the object plane of 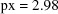
 nm. Note that this pixel size is the fundamental limit of the resolution in the growth direction of the WGA, provided that there is consistent phase retrieval up to the edge of the detector, where the signal (in the 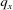 direction) is still sufficiently strong. Specifically, two different supports were tested, denoted by ‘tight’ support and ‘loose’ support, as visualized in Figs. 5 ▸(*c*) and 5 ▸(*d*), respectively. The tight support constraints on the field in the **x** direction were derived from the known parameters of the WGA geometry (design values plus some tolerance, width 647 nm), while the support in the **y** direction (1663 nm) was selected to be much larger than the incoming beam size in the **y** direction.

Contrarily, the loose support corresponds to a rectangle of size 885 and 1663 nm in the **x** and **y** directions, respectively. Note that the primary beam (PB) is not completely absorbed by the WGA and gives a signal in the central maximum on the detector. The corresponding pixels must hence be masked in the projection onto the measurement. To compare the robustness and validity, the ER and HIO phase retrieval algorithms were used, as shown the corresponding object planes in Figs. 5 ▸(*c*) and 5 ▸(*e*). Both were run for 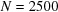 iterations. Fig. 5 ▸(*b*) presents the error metrics for three different cases: tight support using the ER algorithm (red), loose support using the ER algorithm (green) and tight support using the HIO algorithm (light blue). The error metric is computed by (Elser, 2003 ▸)
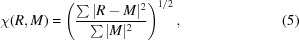
where the summation is over all pixels of the field *R* (reconstructed pattern) and *M* (measured pattern). The error for the tight support using the ER algorithm is smaller than that using the HIO algorithm, which is in line with the general experience with similar phase retrieval problems. Since ER is a local and HIO a non-local optimization, it is often a good strategy to use first HIO and then ER. In the present case, such combinations of HIO and ER were also tested but gave less convincing results than the ER initialized with amplitude data and a flat phase profile. Importantly, for all three reconstructions the beamlets exiting from the WGA can be clearly discerned, as indicated in Figs. 5 ▸(*c*), 5 ▸(*d*) and 5 ▸(*e*). At the same time, the field configurations show differences, which may indicate that the loose support is too ‘weak’ as a constraint. The tight support may also be affected by a small systematic error, since the structure was partially transparent. Owing to the smaller error and most convincing pattern, we primarily compare the results of tight and loose supports using the ER algorithm in the following discussion.

**Figure 6:**
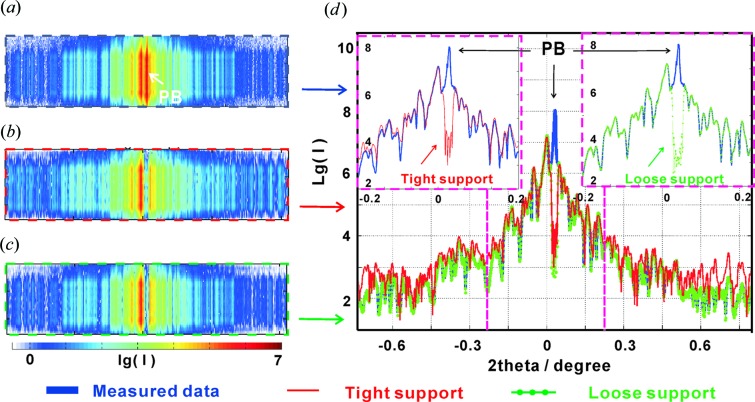
The two-dimensional measured far-field pattern with the transmitted primary beam (PB) of (*a*) the WGA, compared with the reconstructed results of (*b*) the tight support and (*c*) the loose support. (*d*) The corresponding one-dimensional profiles, after integration along the **y** direction: measured far field (blue line), reconstruction with the tight support (red line) and reconstruction with the loose support (green line).

Fig. 6 ▸ allows a comparison of the measured far-field pattern shown in (*a*) with the reconstructed far-field patterns, corresponding to (*b*) the tight and (*c*) the loose support, along with the corresponding line profiles, shown in (*d*). Note that, concerning the experimental data, we have combined the measured data with the same accumulation time from two detector positions (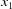 and 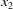) into one (fused) dataset. The three blank regions in the experimental data [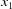 as shown in Fig. 4 ▸(*a*)] are filled with values from the data in 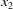, resulting in the combined (fused) data set shown in Fig. 6 ▸(*a*). The profile of the loose support (green line) is highly consistent with the measured data (blue line). For this reason, we select the results from the loose support in the subsequent comparison of field propagation.

**Figure 7:**
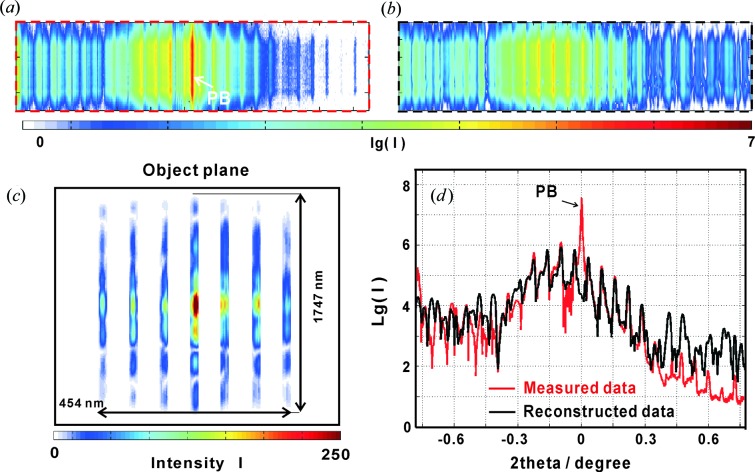
Reconstruction for the WGM (periodic control structure). (*a*) The two-dimensional measured far-field pattern with the transmitted PB, and (*b*) the reconstructed far-field pattern. (*c*) The corresponding reconstructed object plane (size 454 × 1747 nm). (*d*) The integrated one-dimensional profiles for the measured data (red line) and the reconstruction (black line).

Fig. 7 ▸ shows the results for the WGM control structure, again comparing (*a*) the measured and (*b*) the reconstructed diffraction pattern, as well as (*c*) the reconstruction in the object plane. Note that in this case only the tight support gave a satisfactory reconstruction. The support used is also shown in Fig. 7 ▸(*c*) and consists of seven strips of 22 nm width separated by gaps of 50 nm width in a 454 × 1747 nm (**x** and **y** directions) rectangular field. The corresponding one-dimensional far-field pattern in Fig. 7 ▸(*d*) shows a satisfactory agreement between reconstruction (black line) and measurement (red line).

**Figure 8:**
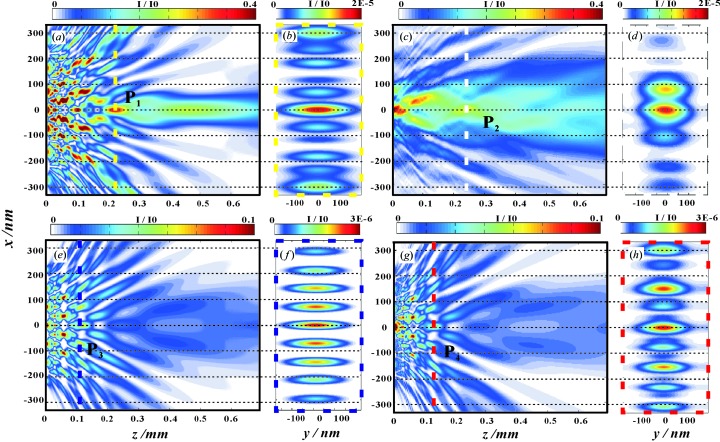
Near-field distribution for (*a*), (*b*) the designed WGA, (*c*), (*d*) the measured WGA, (*e*), (*f*) the control structure WGM in theory and (*g*), (*h*) the measured WGM. (*a*) Design-WGA: one-dimensional FD simulation for the parameters of the designed WGA structure, showing the field in free space behind the exit plane of the WGA. (*b*) Design-WGA: the field distribution in the *P*_1_ 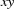 plane, corresponding to the yellow dashed line in (*a*), calculated by full two-dimensional FD simulations. (*c*) Measured-WGA reconstructed by the loose support: one-dimensional free propagation by using the parabolic wave equation in 1 + 1 dimensions (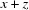 dimensions) (Fuhse & Salditt, 2005 ▸), starting from the reconstructed near-field pattern of the WGA [reconstruction data corresponding to Fig. 5 ▸(*d*)]. (*d*) Measured-WGA reconstructed by the loose support: the field distribution in the *P*_3_ 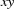 plane, corresponding to the white dashed line in (*c*), calculated by the parabolic wave equation in 2 + 1 dimensions (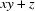 dimensions). (*e*) Design-WGM: one-dimensional FD simulation for the parameters of the designed WGM structure, showing the field in free space behind the exit plane of the WGM. (*f*) Design-WGM: the field distribution in the *P*_3_ 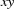 plane, corresponding to the dark-blue dashed line in (*e*), calculated by full two-dimensional FD simulations. (*g*) Measured-WGM: one-dimensional free propagation by using the parabolic wave equation in 1 + 1 dimensions, starting from the reconstructed near-field pattern of the WGM [reconstruction data corresponding to Fig. 7 ▸(*c*)]. (*h*) Measured-WGM: the field distribution in the *P*_4_ 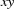 plane, corresponding to the red dashed line in (*g*), calculated by the parabolic wave equation in 2 + 1 dimensions.

Next, we compute the near-field propagation along the optical axis *z*, starting from the complex-valued field in the reconstruction plane, and compare this with the simulation according to the (ideal) design values. To this end, we carry out FD simulations in two different dimensional settings: Simulations denoted as 1 + 1 dimensional have one dimension along the optical axis **z** and one dimension **x** orthogonal to the optical axis parallel to the normal vector of the thin-film interfaces. Simulations denoted as 2 + 1 dimensional take into account both dimensions orthogonal to the optical axis, *i.e.* also the direction **y**, in which the planar waveguide is translationally invariant. Fig. 8 ▸(*a*) shows the designed (ideal) field distribution obtained from the FD simulations (simulated in 1 + 1 dimensions), with the yellow dashed line indicating the quasi-focus in plane *P*_1_ (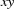 plane) at a distance *z* = 226.0 µm. Fig. 8 ▸(*b*) shows the pattern in the *P*_1_ plane (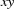 plane) as calculated in 2 + 1 dimensions. These results can be compared with the experimental reconstruction results with the loose support in Figs. 8 ▸(*c*) and 8 ▸(*d*), based on using the parabolic wave equation (Fuhse & Salditt, 2005 ▸). The quasi-focal point is at 247.1 µm in the *P*_2_ plane (white dashed line). We see that the field distribution is only in qualitative agreement. This can be expected from the deviations of the layer parameters from the design values (see Table 2 ▸). In addition, the experimental setting was not perfect, since the incoming beam intensity was probably not constant over the entire structure range, as indicated by the reconstructions of the exit wave. Importantly, however, the quasi-focal spots are still observed in the experimental result. The FWHM of the quasi-focal spot in the *P*_2_ plane is 45.0 nm (Fig. 8 ▸ *d*) along **x**, which is not much larger than the design value of 22.0 nm (Fig. 8 ▸ *b*). Furthermore, as desired, the field distribution of the WGA is significantly different from the WGM control structure as shown in Figs. 8 ▸(*e*)–8 ▸(*h*). In this case (WGM), the field distribution is again calculated from the WGM experimental values (field reconstruction with tight support). The near-field pattern (Figs. 8 ▸ *g* and 8 ▸ *h*) is very close to the simulated one (Figs. 8 ▸ *e* and 8 ▸ *f*). Note that the intensities are lower than for the WGA, owing to the longer working length 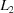 and correspondingly higher absorption. Importantly, the interference patterns have no obvious central peak as for the WGA. We conclude that the reconstructed field pattern for the WGA (both in 1 + 1 dimensions and in 2 + 1 dimensions) shows the characteristic features of the design structure, supporting the concept of near-field control by variation of guiding layer thickness.

## 5. Discussion and conclusion   

In summary, we have reconstructed the near-field distribution of an Ni/C X-ray waveguide array (WGA) from the measured far-field data. To this end, we have used two different supports (the tight support and the loose support). Phase retrieval of one-dimensional structures is known to be problematic. Despite the fact that the experiment has used a focused beam and a two-dimensional detection scheme, and hence falls into the (nominal) category of two dimensional, the variation of the signal is essentially one dimensional. For this reason we had anticipated that we would require as much support information as possible, and have therefore used the strong *a priori* information of position of the seven waveguide channels (the beamlets). However, in the case of the WGA a tight support did not turn out to be necessary, and the loose support actually gave smaller errors in the reconstruction. The reconstruction results are quite robust. The coarse pattern of the reconstructed field was similar in both cases. Contrarily, for the periodic WGM, the tight support turned out to be necessary, which is not surprising given the known difficulty associated with phase retrieval of periodic structures. Therefore, the constraints have to be tightened. Note that we also performed one-dimensional phase retrieval based on detector data summed over the columns. As expected, these reconstructions were less stable.

Using the two-dimensional phase retrieval, we could validate the concept of tailored near-field distributions, put forward before on the basis of analytical theory and numerical simulations. According to this concept, the multi-beam interference pattern is controlled by variation of both seven C guiding layer thicknesses and eight Ni cladding layer thicknesses in the experiment. This leads to beam intensity modulations in the free space behind the waveguide exit, which are distinctly different from those obtained for a WGM with seven constant C guiding layer thicknesses and eight constant Ni cladding layer thicknesses. In particular, quasi-focal spot sizes in the sub-50 nm range can be generated. In future, such tailored near fields exhibiting large structural diversity could be used for coherent imaging, for example by ptychography (Thibault *et al.*, 2008 ▸; Maiden & Rodenburg, 2009 ▸; Guizar-Sicairos *et al.*, 2008 ▸), which has been shown to benefit from a highly structured illumination wavefield. Note that, not only for imaging applications but also as a more powerful probe reconstruction for inspection of the WGA near field, ptychography is an obvious extension for future work. Finally, we suggest that future generalizations of the WGA concept could include design of twin peaks for differential phase contrast, or emission of radiation directed away from the optical axis (off-axis), similar to the optics of distributed antennas in other spectral ranges.

## Figures and Tables

**Table 1:** The theoretical WGA and WGM designed layer thickness

Layer No.	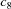	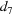	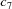	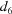	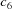	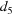	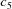	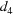	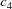	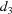	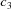	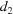	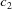	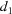	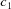	Sub
Layer name	Ni top	C	Ni	C	Ni	C	Ni	C	Ni	C	Ni	C	Ni	C	Ni	Si Sub

Waveguide array (WGA)																
Layer thickness (nm)	50.0	15.7	92.3	17.2	72.8	17.8	54.2	18.0	54.2	17.8	72.8	17.2	92.3	15.7	50	

Periodic waveguide multilayer (WGM)																
Layer thickness (nm)	50.0	18.0	54.0	18.0	54.0	18.0	54.0	18.0	54.0	18.0	54.0	18.0	54.0	18.0	54.0	

**Table 2:** Design parameters and averaged layer thickness as determined by TEM for the WGA structure

Layer No.	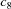	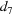	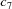	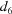	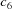	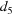	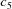	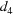	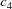	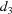	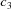	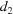	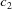	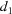	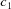	Total thickness (nm)
Layer name	Ni	C	Ni	C	Ni	C	Ni	C	Ni	C	Ni	C	Ni	C	Ni	

Theoretical design results																
Layer thickness (nm)	50.00	15.70	92.30	17.20	72.80	17.80	54.20	18.00	54.20	17.80	72.80	17.20	92.30	15.70	50.00	658.00

TEM reading averaged results, error bar ±0.45 nm																
Layer thickness (nm)	51.36	15.40	95.60	16.87	76.16	17.19	57.05	17.60	57.10	17.15	76.60	16.16	96.37	14.62	52.29	677.52
